# Thirdhand smoke beliefs, exposure status and associated factors among young people in China: A cross-sectional study

**DOI:** 10.18332/tid/171352

**Published:** 2023-10-13

**Authors:** Qinyi Guan, Jianrong Mai, Kaisheng Teng, Zhihong Liu, Lina Lin, Ling Zhou, Tingfen Huang, Xiaoyu Tan, Xinying Sun

**Affiliations:** 1 School of Public Health, Guangxi Medical University, Nanning,China; 2 School of Nursing, Guangzhou Xinhua University, Guangzhou, China; 3 School of Public Health, Peking University, Beijing, China

**Keywords:** thirdhand smoke, exposure status, beliefs, young people, associated factors, China

## Abstract

**INTRODUCTION:**

Thirdhand smoke is an emerging threat to global public health. There is no research on young people's exposure to thirdhand smoke in China. This study aims to investigate the exposure status and beliefs of thirdhand smoke among young Chinese people and provide a reference for policy regarding thirdhand smoke.

**METHODS:**

Data from the 2022 Chinese Resident Psychological and Behavioral Survey were used to select young people aged 15–24 years. A total of 11781 subjects were included in this study. Demographic information, beliefs about thirdhand smoke, and exposure status to thirdhand smoke were investigated.

**RESULTS:**

Among the participants, 47.8% reported being exposed to thirdhand smoke (males: 49.1%, females: 47.0%). Young people living in urban areas (AOR=0.84, 95% CI: 0.77–0.91, p<0.001) and those with a monthly family income >12000 RMB (AOR=0.81, 95% CI: 0.71–0.92, p=0.001) were less likely to be exposed to thirdhand smoke. Young people with junior high school education or higher, and current or former smokers, were more likely to be exposed to thirdhand smoke. There is room for improvement in the beliefs about thirdhand smoke among young people.

**CONCLUSIONS:**

Thirdhand smoke exposure is an issue that should be addressed in public health policy. Young people with low income and current or former smokers are populations that should be mainly focused on in public education and prevention work on thirdhand smoke.

## INTRODUCTION

Tabacco hazards have always been a major global public health issue, causing the deaths of over 8 million people each year, including 1.2 million non-smokers exposed to secondhand smoke^1^. More than 300 million people in China are tobacco users, and every year 100000 non-smokers die from secondhand smoke^2^. The harm of secondhand smoke is not less than that of direct smoking. Since the World Health Organization released the Framework Convention on Tobacco Control in 2005, there has been a global decline in exposure to secondhand smoke, with the number of non-smoking adults exposed to secondhand smoke decreasing significantly^3^. A survey conducted in Scotland showed that the number of non-smoking adults who were exposed to secondhand smoke had significantly decreased^4^. However, in-depth research on tobacco hazards has revealed thirdhand smoke as an emerging threat to global public health. Some scholars have pointed out that the carcinogenic substance content in thirdhand smoke is 10 times higher than in secondhand smoke^5^. Thirdhand smoke was first introduced by a pediatrician in 2009 and refers to the compounds (such as nicotine, nitrite, etc.) emitted in secondhand smoke that adhere to the surfaces of objects (such as furniture, clothing, and decor) in the environment, and produce new harmful substances that can be released into the environment through a series of chemical reactions^5,6^. Evidence shows that thirdhand smoke not only changes and damages DNA structure but also increases non-smokers’ cancer incidence rate and causes cytotoxicity that affects mouse development^7^. Wen et al.^8^ pointed out that thirdhand smoke is potentially one of the risk factors for cervical cancer in Chinese women, and they noted a dose-response relationship between the exposure time to thirdhand smoke and the risk of cervical cancer. Thirdhand smoke is a very difficult indoor secondary toxin, and even opening windows for ventilation cannot eliminate it, and it can linger indoors for a long time^9,10^. In addition, the concentration of toxic substances in thirdhand smoke will continue to increase over time, which means that the harm of thirdhand smoke to human health will continuously increase over time^11^. Most of the reported studies on thirdhand smoke, both domestic and international, were conducted among children, parents, and medical personnel, and these studies were divided into mechanism-of-harm research and belief-survey research^12,13^. A survey of medical personnel showed that more than two-thirds of medical personnel believed that thirdhand smoke was not receiving enough attention^14^. The study of Thomas et al.^15^ indicated that although smoking bans in homes reduced the level of thirdhand smoke, prohibiting indoor smoking may not be sufficient to protect children from its effects in households with high levels of smoking.

Thirdhand smoke can harm people’s health and has become a new trend in research on tobacco hazards^10^. However, there is currently little research on young people’s thirdhand smoke exposure in China. Therefore, based on the 2022 Chinese Resident Psychological and Behavioral Survey, which is a large, nationally representative, and cross-sectional survey. This study aims to investigate the beliefs and exposure status of thirdhand smoke and the relationship between them among young Chinese people and analyze the associated factors. We hope to provide a theoretical basis for implementing educational campaigns on thirdhand smoke.

## METHODS

### Study setting and participants

**Figure 1:**
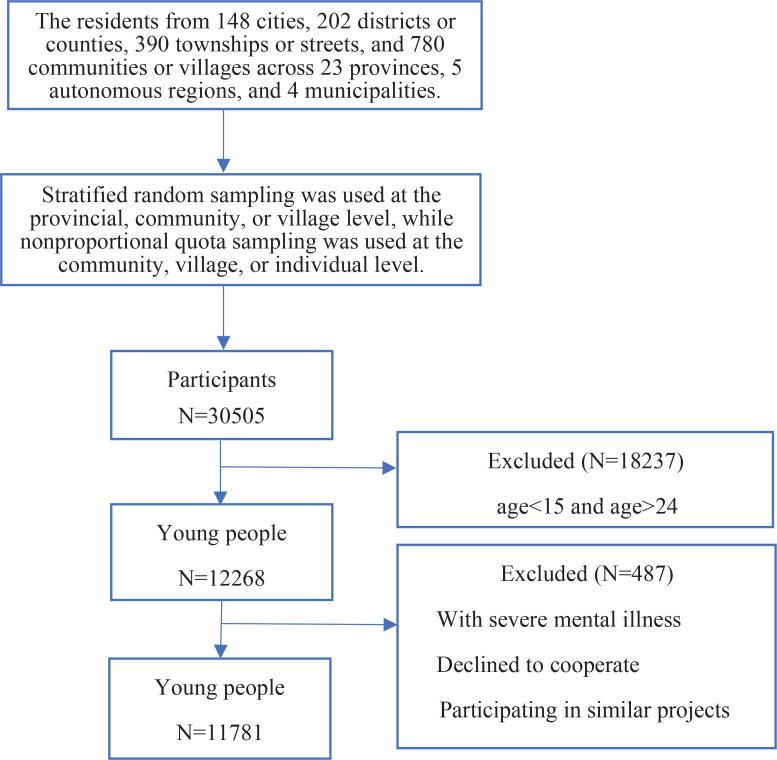
Flow chart of participant selection

A multi-stage random sampling method was used to recruit participants from 20 June to 31 August 2022 in this cross-sectional study. The research covered 148 cities, 202 districts or counties, 390 townships or streets, and 780 communities or villages in 23 provinces, 5 autonomous regions, and 4 municipalities. Stratified random sampling was employed at the provincial, community, or village level, whereas non proportional quota sampling was utilized at the community, village, or individual level. The interviewers were invited to a community healthcare center or community neighborhood committee to complete the questionnaire. A unique web link for the electronic questionnaire was distributed face-to-face by trained investigators to voluntary participants in each city where the survey was conducted. If in-person surveys were not feasible due to COVID-19 restrictions, investigators could use platforms such as Tencent Conference and WeChat video for one-on-one online video surveys. Inclusion criteria: 1) age 15–24 years; 2) Chinese residents (having not been away for more than one month); 3) voluntary participation and signing of informed consent; 4) ability to complete the online questionnaire independently or with the assistance of investigators; 5) holding Chinese citizenship; 6) understanding the meaning conveyed by each item on the questionnaire. Exclusion criteria were : 1) participation in similar studies; 2) suffering from severe mental illness; and 3) refusal to collaborate. A total of 30505 valid questionnaires were collected. This study used data from individuals aged 15–24 years. A total of 11781 young people were included in this study (Figure 1).

### Definitions of variables

Thirdhand smoke exposure refers to staying in an area where someone has smoked at least one day in the past 7 days^16,17^. In this study, participants who stayed in an area where someone had smoked at least one day in the past 7 days were classified as the exposed group, while the rest were classified as the non-exposed group^16,17^. This study excluded secondhand smoke exposure by asking respondents whether people smoked in front of them.

The thirdhand smoke exposure prevalence was defined as the percentage of participants who reported staying in an area where a person had smoked during the past 7 days to the total number of participants in this study^17,18^.

### Measurements

*Sociodemographic information*

Information included gender, ethnicity, education level, residence, marital status, monthly family income (in RMB), and smoking status.

*Beliefs about thirdhand smoke*

Adapted from the thirdhand smoke beliefs scale developed by Regine et al.^19^, this questionnaire consisted of 7 items rated on a 4-point Likert scale, ranging from ‘strongly disagree’ (1 point) to ‘strongly agree’ (4 points), with a total score ranging 7–28. A higher score indicates greater awareness of thirdhand smoke. The Cronbach’s alpha coefficient for this questionnaire was 0.944, indicating good reliability.

*Thirdhand smoke exposure*

Participants were asked: 1) whether people smoked in areas where they lived/worked during the past 7 days; 2) in which areas people smoked where they lived/worked during the past 7 days; and 3) how long they stayed in areas where people had smoked during the past 7 days.

### Statistical analysis

**Figure 2:**
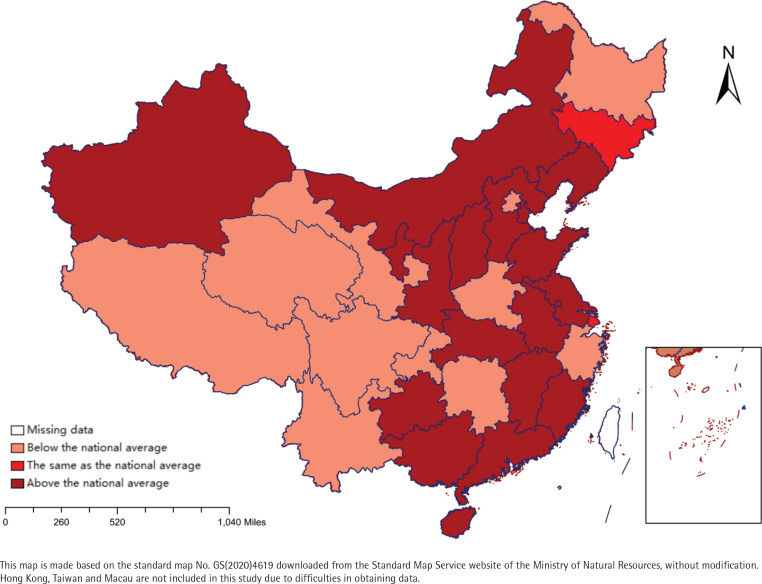
Map of thirdhand smoke exposure among young people in China

This study used SPSS 26.0 software for statistical analysis of the data. Quantitative data were presented as mean and standard deviation, and qualitative data were presented as frequency and percentage. Firstly, the group comparison was conducted using the χ^2^-test and then, using exposure to thirdhand smoke as the dependent variable and the score of thirdhand smoke beliefs as independent variables, adjusting for age and variables that were found to be statistically significant in the χ^2^-test. A binary logistic regression analysis was conducted to identify factors influencing thirdhand smoke exposure among young people. The independent variables included general demographic data, variables identified by single-factor analysis, and scores from the thirdhand smoke awareness questionnaire, with specific variable assignments shown in Table 1. Finally, the results (adjusted) of binary logistic regression analysis were imported into Rstudio 4.2.2, and a forest plot was generated using the forest plot package to visualize the data results. In addition, we used Excel to calculate the thirdhand smoke exposure provinces among young people in various provincial administrative regions in China and then imported the data results into Arcgis10.8 software. We compared the thirdhand smoke exposure of young people in different cities with the national average prevalence (47.8%). We created a current situation map of thirdhand smoke exposure among Chinese young people in the provinces, dividing them into three categories: higher than the national level, equal to the national level, and lower than the national level (Figure 2).

**Table 1:** Assignment of variables for the binary logistic regression

*Variables*	*Assignment*
Thirdhand smoke exposure	0: No, 1: Yes
Gender	1: Male, 2: Female
Nationality	1: Han, 2: Minority
Education level	1: Primary school and lower, 2: Middle school 3: High school, 4: Junior college 5: Bachelor’s degree or higher
Residence	1: Rural, 2: Urban
Marital status	1: Unmarried, 2: Other
Smoking status	1: Non-smoker, 2: Smoker, 3: Ex-smoker
Family monthly income (RMB)	1: <5000; 2: 5000–12000; 3: >12000

The significance test was two-sided, and the significance level for all analyses was α=0.05. RMB: 1000 Chinese Renminbi about US$160.

## RESULTS

### Study sample characteristics

The survey included a total of 11781 participants, of whom 4838 were male (41.1%), and 6943 were female (58.9%). Among them, 10675 people (90.6%) did not smoke, 892 people (7.6%) were current smokers, and 214 people (1.8%) were former smokers. In terms of education level, 1.1% had primary school education or lower, 5.9% had junior high school education, 31.2% had vocational/high school education, 9.8% had associate degree education, and 52.0% had undergraduate or higher education. In terms of residence, 24.3% were rural residents, and 75.7% were urban residents.

### The score of thirdhand smoke beliefs

Table 2 presents the overall score for awareness of thirdhand smoke beliefs in this survey which was 21.45 ± 4.80 points, with scores above 3.0 for all items except items 4 and 6, which had lower scores. Item 1 (‘Being in a room where someone smoked yesterday can harm the health of adults or children’) rated the highest average score, while item 6 (Particles from surfaces contaminated by smoke can enter the body through the skin’) had the lowest average score.

**Table 2:** Young people’s response score to each of the 7-items assessed by the Beliefs About Thirdhand Smoke (BATHS) scale, China (N=11781)

*Items*	*Mean ± SD*
1. Breathing air in a room today where people smoked yesterday can harm the health of infants, children, and adults.	3.25 ± 0.78
2. Particles in rooms where people smoked yesterday can cause cancer.	3.04 ± 0.81
3. Smoke particles can remain in a room for weeks and months.	3.07 ± 0.80
4. Smoke particles get absorbed into furniture and walls.	2.99 ± 0.82
5. After smoking a cigarette, smoke particles on skin, hair, and clothing can be passed on to others through touch.	3.03 ± 0.79
6. After touching surfaces where cigarette smoke has settled, particles can enter the body through the skin.	2.97 ± 0.83
7. Opening windows or using air conditioners does not eliminate all smoke particles in a room.	3.10 ± 0.76
**Total points**	21.45 ± 4.80

### Thirdhand smoke exposure

The surveyed individuals reported being exposed to thirdhand smoke in the past 7 days at home (41.1%), in transportation (10.3%), at work (15.8%), at school (13.8%), in restaurants (11.6%), and other environments (12.6%). The prevalence of thirdhand smoke exposure among Chinese young people was 47.8%. Among them, 13 provinces (Shandong, Guizhou, Hainan, Guangdong, Liaoning, Anhui, Jiangxi, Hebei, Hubei, Shaanxi, Jiangsu, Shanxi, and Fujian), 4 autonomous regions (Guangxi Zhuang Autonomous Region, Ningxia Hui Autonomous Region, Xinjiang Uygur Autonomous Region, and Inner Mongolia Autonomous Region), and Tianjin Municipality, had higher prevalences of thirdhand smoke exposure among young people than the national average level. Meanwhile, Jilin Province and Shanghai Municipality had equal to the national average level, and in all other areas (excluding Hong Kong, Macao, and Taiwan), the thirdhand smoke exposure prevalence among young people was lower than the national average level (Figure 2).

### Analysis of associated factors of thirdhand smoke exposure

Although all participants had a certain level of belief about thirdhand smoke, it was relatively basic knowledge. In this large-scale, nationally representative study in China, 47.8% of the participants were exposed to thirdhand smoke. Compared with non-smokers, current smokers had the highest risk of exposure to thirdhand smoke, followed by ex-smokers. The education level, beliefs of thirdhand smoke, and living in rural areas, were associated with a greater risk of exposure to thirdhand smoke.

Table 3 presents the results of the χ^2^-test. There were statistically significant differences (p<0.05) in thirdhand smoke exposure among individuals according to gender, ethnicity, education level, residence, smoking status, and monthly household income. In addition, there were no statistically significant differences in thirdhand smoke exposure among individuals in the Central, North, East, South, Northeast, Southwest, and Northwest regions of China.

**Table 3:** Charcteristics of participants and differences in thirdhand smoke exposure prevalence among young people in China (N=11781)

*Variables*	*Number of people exposed to thirdhand smoke n*	*Thirdhand smoke exposure prevalence %*	*χ^2^*	*p*
**Gender**			5.217	0.022
Male	2375	49.1		
Female	3260	47.0		
**Nationality**			6.849	0.009
Han	4916	47.4		
Minority	719	51.1		
**Religious belief**			0.970	0.325
Yes	98	44.5		
No	5537	47.9		
**Education level**			44.846	<0.001
Primary school and lower	33	26.0		
Middle school	286	40.9		
Vocational secondary school/ High school	1179	48.4		
Junior college	595	51.5		
Bachelor’s degree or higher	2942	48.0		
**Residence**			21.623	<0.001
Rural	1480	51.6		
Urban	4155	46.6		
**Marital status**			0.018	0.892
Unmarried	5511	47.8		
Other	124	48.2		
**Smoking status**			211.861	<0.001
Non-smoker	4876	45.7		
Smoker	616	69.1		
Ex-smoker	143	66.8		
**Family monthly income** (RMB)			10.605	0.005
<5000	3523	48.9		
5000–12000	1625	46.9		
>12000	487	44.0		

RMB: 1000 Chinese Renminbi about US$160.

**Figure 3:**
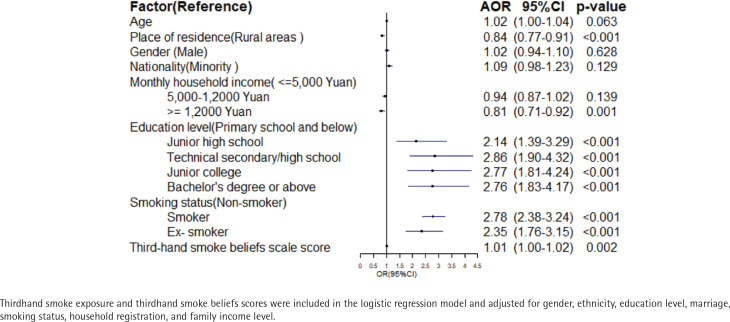
Binary logistic regression analysis of thirdhand smoke exposure in young people in China

Figure 3 presents the result of binary logistic regression. Compared with young people who live in rural areas, the odds of exposure to thirdhand smoke were lower among those living in urban areas (AOR=0.84; 95% CI: 0.77–0.91). An income of >12000 RMB (AOR=0.81; 95% CI: 0.71–0.92) was a protective factor for exposure to thirdhand smoke. An association was observed between exposure and beliefs in thirdhand smoke (AOR=1.01; 95% CI: 1.00–1.02). Interestingly, education level and beliefs in thirdhand smoke are risk factors for exposure to thirdhand smoke. Furthermore, compared with non-smokers, current smokers (AOR=2.79; 95% CI: 2.38–3.24) and ex-smokers (AOR=1.76; 95% CI: 1.76–3.15) were more likely to be exposed to thirdhand smoke among Chines young people.

## DISCUSSION

Although all participants had a certain level of belief about thirdhand smoke, it was relatively basic knowledge. In this large-scale, nationally representative study in China, 47.8% of the participants were exposed to thirdhand smoke. Compared with non-smokers, current smokers had the highest risk of exposure to thirdhand smoke, followed by ex-smokers. The education level, beliefs of thirdhand smoke, and living in rural areas, were associated with a greater risk of thirdhand smoke exposure.

Most people know that tobacco smoke is harmful to health but are unaware that the particles can harm the body by entering it through the skin. This is the reason why although tobacco hazard education has achieved certain results, there is still significant room for improvement. A survey of Shanghai primary school students in 2019 on thirdhand smoke hazard awareness found that the item ‘being in a room where someone smoked yesterday can harm the health of adults or children’ had the highest average score, consistent with the results of this study^20^. Another survey found that only 24.5% of participants knew that particles from surfaces contaminated by smoke could enter the body through the skin^21^. A qualitative study among young people in Tianjin, China, showed that although participants were aware of the risks of smoking, they had limited knowledge of the health effects associated with it^22^.

The overall thirdhand smoke exposure prevalence for young people in this survey is relatively higher than in other research. A study from the United States showed that among a sample of 17300 children, 2278 were exposed to thirdhand smoke, resulting in a thirdhand smoke exposure prevalence of 13.2%^23^. The MPOWER tobacco control strategy proposed by the World Health Organization in 2008 provides important references and guidance for China’s implementation and promotion of smoke-free policies in public places^24^. Homes had the highest proportion of thirdhand smoke exposure (41.1%), significantly higher than other different settings (10–16%) in our study. Although China has laws and regulations prohibiting smoking in certain public places, smoking behavior within households is still uncontrolled^25^. Previous research has indicated that households are the main source of children’s secondhand smoke exposure, and thirdhand smoke, as a secondary toxicant derived from secondhand smoke, is consistent with the main source of adolescent thirdhand smoke exposure in this survey^26^. Continued exposure to thirdhand smoke increases the risk of disease, and creating smoke-free homes can reduce the probability of adolescent thirdhand smoke exposure^17^. Currently, most studies on smoke-free homes indicate that they can reduce secondhand smoke exposure, but few studies have focused on the relationship between smoke-free homes and thirdhand smoke^27^. It is worth noting that thirdhand smoke exposure in provinces, in workplaces, and schools, is slightly higher than in transportation, restaurants, and other environments. Typically, workplaces and schools have more stable personnel than transportation, restaurants, and other environments, which makes it easier for ‘passive smoking behaviors to occur, influencing the likelihood of adolescent thirdhand smoke exposure in these settings. In the Chinese social context, ‘passive smoking behavior’ is considered a polite way of conversation that can strengthen social connections through ‘passing cigarettes’^28^.

The risk of thirdhand smoke exposure for young people living in rural areas is higher than for urban residents. According to one study, the number of smokers in rural China exceeds 200 million, twice that of urban areas^29^. This study confirms our results from a probabilistic perspective. In addition, urban areas in China have more abundant economic, educational, and medical resources than rural areas, which is associated with a lower probability of thirdhand smoke exposure for urban residents. Studies have shown that the smoking prevalence among elderly urban residents is significantly lower than that of rural elderly, and older urban residents have a higher correct score when answering all tobacco-related questions compared to rural elderly^30^. Young people with higher education are more likely to believe that thirdhand smoke will continue to exist and affect children’s health^20^. Lei et al.^31^ pointed out that men with higher education tend to quit smoking faster than those with lower education level. However, education level is associated with risk of exposure to thirdhand smoke among young people in our study. Taking into account the age range of the study participants, it is quite difficult to consider education level as a potential predictor. Not all the participants have achieved their full possible education. Parental education is usually more informative in such cases. Unfortunately, we did not consider parental education at first. Individual tobacco use has been consistently associated with family income. Previous studies have shown that the lower the family income, the more likely tobacco products will be used^32,33^. Similarly, family income is inversely proportional to the probability of adolescent thirdhand smoke exposure in our study. In this study, the probability of young people who smoke being exposed to thirdhand smoke was higher than that of non-smokers because smokers’ level of awareness of tobacco hazards is often lower than that of non-smokers, making them more exposed to thirdhand smoke environments^34^. Because thirdhand smoke is a relatively new concept, the association between young people’s knowledge of thirdhand smoke and their exposure is weak.

### Limitations

Because thirdhand smoke exposure is a relatively new field, there is currently no unified indicator for measuring thirdhand smoke exposure. While the study tried to rule out secondhand smoke exposure as much as possible by asking respondents if people smoked in front of them, it cannot be ruled out completely. Therefore, the relevant indicators in this article are self-inducted based on references to similar studies. This study was designed as a cross-sectional study, so the exact causal relationship cannot be determined. Further research is needed in the future. Due to the difficulty of data collection, this study did not include data from Hong Kong, Macau, and Taiwan regions, which limits the generalizability of the results. In addition, China has a vast territory, and smoking culture varies from province to province. This study did not take this issue into account. Similarly, due to the diverse social contexts in different countries, the generalizability of this study to other countries is limited. Future research will need to investigate the relationship between smoking culture and the likelihood of adolescent thirdhand smoke exposure.

## CONCLUSIONS

This nationally representative large-scale study showed that among young Chinese people, the prevalence of exposure to thirdhand smoke is high, and the beliefs about thirdhand smoke need to be strengthened. People need to pay attention to the health hazards of thirdhand smoke. The thirdhand smoke awareness score of young people indicates that when conducting scientific dissemination of thirdhand smoke, relevant departments need to make the content more detailed and carry out targeted scientific dissemination work. Tobacco control policies should be different for adults and young people^22^. Low income and smoking/quitting young people are the focus of thirdhand smoke dissemination. In the future, the government must accelerate the promotion and creation of smoke-free homes to protect the health of young people. The dissemination, prevention, and elimination of thirdhand smoke will be one of the key elements in future tobacco control work. This study provides empirical evidence for the prevention and elimination of thirdhand smoke.

## Data Availability

The data supporting this research are available from the authors on reasonable request.
